# Overnight Stay in the Emergency Department and In-Hospital Mortality Among Elderly Patients: A 6-Year Follow-Up Italian Study

**DOI:** 10.3390/jcm14092879

**Published:** 2025-04-22

**Authors:** Andrea Fabbri, Ayca Begum Tascioglu, Flavio Bertini, Danilo Montesi

**Affiliations:** 1 Emergency Department, Local Health Agency of Romagna, Presidio Ospedaliero Morgagni-Pierantoni, via C Forlanini 34, 47121 Forlì, Italy; 2 Department of Computer Science and Engineering, University of Bologna, Mura Anteo Zamboni 7, 40126 Bologna, Italy; aycabegum.tascioglu2@unibo.it (A.B.T.); danilo.montesi@unibo.it (D.M.); 3 Department of Mathematical, Physical and Computer Sciences, University of Parma, Parco Area delle Scienze 53/A, 43124 Parma, Italy; flavio.bertini@unipr.it

**Keywords:** emergency department, older age, predictors, mortality, in-hospital beds

## Abstract

**Background/Objectives:** Due to challenges in securing hospital beds, elderly patients may face prolonged emergency department (ED) stays. Recent studies have highlighted an association between ED overnight stays (EDOSs) before admission and increased mortality. This study aimed to evaluate the potential impact of EDOSs on mortality among elderly patients awaiting a regular bed in a standard hospital ward. **Methods:** This monocentric, retrospective study included subjects ≥ 75 years who required urgent hospitalization between 2017 and 2022. Two groups were compared: patients hospitalized between 00:00 and 08:00 following an ED overnight stay (EDOS group), and those admitted directly to conventional medical units between 08:00 and 00:00 (Ward group). The primary outcome was in-hospital mortality 30 days after ED visit. **Results**: Among the 20,009 patients included (median age: 85 years [IQR: 80–89]), 3064 (15.3%) belonged to the EDOS group, while 16,945 (84.7%) were in the Ward group. In-hospital mortality occurred in 3020 cases (15.1%), with no significant differences observed between the groups. The variables identified by the logistic model as predictors of mortality included age > 85 years, Charlson Comorbidity Index (CCI) ≥ 5, National Early Warning Score (NEWS) > 6 at arrival, infectious diseases, respiratory diseases, and circulatory system diseases, yielding an accuracy of 0.700 ± 0.007. EDOS while awaiting inpatient beds was not a predictor of mortality. **Conclusions**: The results of our study did not show an association between mortality and EDOS, even when considering the large sample size collected over 6 years and the varying percentages of patients awaiting hospital beds.

## 1. Introduction

### 1.1. Background/Rationale

Optimal resource allocation in emergency departments (EDs) is essential for delivering high-quality care, especially for patients requiring urgent hospitalization. Overcrowding and prolonged ED stays are major factors that negatively affect health assistance and quality of care, potentially influencing in-hospital mortality [1,2,3].

A prolonged stay in the ED is a source of dissatisfaction for both patients and their families [4]. However, this indicator alone is not sufficient to fully evaluate the quality of care. Combining the long time spent in the ED and the occurrence of unfavorable events during hospitalization could be relevant because it could contribute to improving the quality of care, and therefore affect the prognosis of patients.

Recent studies have indicated that extended stays in the ED, particularly spending an entire night waiting for an in-hospital bed, are associated with higher hospital mortality among elderly patients [5,6,7]. For instance, a French study [6] identified a link between ED overnight stays (EDOSs) and increased mortality in this population. Conversely, a similar Spanish study [8] failed to replicate these findings.

### 1.2. Objectives

Our objective was to evaluate whether overnight stays in the ED were associated with increased mortality by analyzing a large dataset spanning six years. To test this hypothesis, we examined the impact of a progressively increasing percentage of overnight stay patients using balancing techniques and random sampling.

## 2. Methods

### 2.1. Study Design, Setting, and Participants

**Figure 1:**
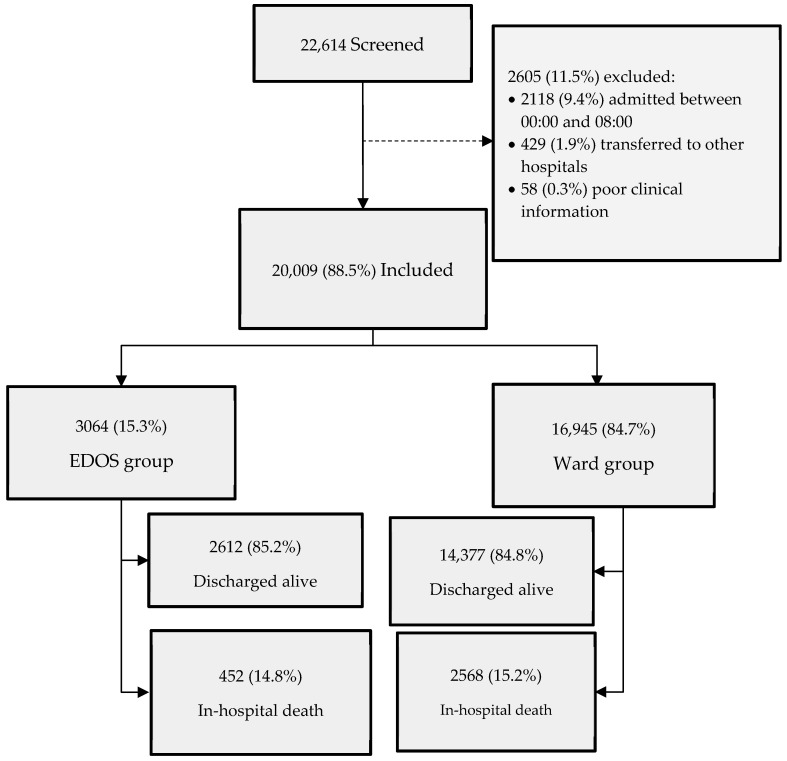
Flow diagram of study patients during the study period of 2017–2022.

The data used in this analysis were collected from the official registry of the local health agency of Romagna, Forlì, Italy, between 1 January 2017 and 31 December 2022. An initial screening of 22,614 patients aged ≥ 75 years and admitted to the ordinary ward was performed. Following the initial screening, a total of 2605 cases (11.5%) were excluded due to missing clinical information (N = 58; 0.2%), transfer to another hospital (N = 429; 1.9%), and admissions between 00:00 and 08:00 AM (N = 2118; 9.4%) (Figure 1).

The final analysis was performed on a sample of 20,009 patients by comparing two groups: the first group included patients admitted before midnight (Ward group), while the second group included patients admitted after 8:00, who had spent at least one night in the emergency room (EDOS group) (Figure 1). All patients were followed up for 30 days.

### 2.2. Variables

The primary outcome measure for the logistic model was in-hospital mortality within 30 days after ED visit. Due to the imbalance in the number of cases for the study variables (EDOS), we tested whether random sampling with increasing percentages of EDOS events would affect the results, even with a reduction in sample size. The RandomUnderSampler from the imbalanced-learn Python library was used to balance the dataset. We explored various sampling strategies by adjusting the sampling percentage, which varied from 0.1 to 1.0 in increments of 0.1. This parameter controls the target ratio of the number of samples in the minority class relative to the number of samples in the majority class after resampling [9]. Most baseline patient characteristics, including age and sex, were collected at the time of ED registration. Vital signs—specifically systolic blood pressure (SBP), heart rate (HR), respiratory rate (RR), and temperature—were recorded upon arrival to calculate the NEWS [10]. The NEWS was considered as a categorical variable (0–4 low risk, 5–6 medium risk, >6 high risk). The CCI [11] was calculated based on free-text patient reports extracted during the ED visit. In the analyses, the CCI was considered in the following categories (1–2 mild, moderate 3–4, severe ≥ 5). An internal validation of the comorbidity extraction algorithm was performed using 500 patient reports annotated by a medical expert, achieving over 90% accuracy for each comorbidity. The CCI formula considered was as follows: myocardial infarction, congestive heart failure, peripheral vascular disease, cerebrovascular disease, dementia, chronic pulmonary disease, connective tissue disease, ulcer disease, mild liver disease, and diabetes (1 point); hemiplegia, moderate or severe renal disease, diabetes with end-organ damage, any tumor, leukemia, and lymphoma (2 points); moderate or severe liver disease (3 points); and metastatic solid tumor and AIDS (6 points). Additionally, age adjustments were applied: 1 point was added for each decade over 40 years (e.g., 50 years = +1, 60 years = +2, 70 years = +3, etc.), with these “age points” added to the total CCI score [12]. Length of stay (LoS) and EDOS were calculated as the difference between the entry and exit times.

### 2.3. Statistical Methods

Data analysis was conducted on a cohort of 20,009 patients aged ≥ 75 years. The study compared two groups: those admitted to a hospital ward after spending the entire night in the ED and those admitted between 08:00 and 00:00. Patients admitted between 00:00 and 08:00 were excluded from this comparison. Continuous variables were reported as either the mean (standard deviation, SD) or median [Interquartile range, IQR]. Categorical variables were summarized as counts and percentages. Differences in the patient characteristics, along with the corresponding 95% confidence intervals (CIs), were calculated. A *p*-value of less than 0.05 was considered statistically significant for all analyses.

The primary outcome measure was in-hospital mortality, comparing the EDOS group with the Ward group. A prognostic model tested several variables, three of which reflected the patients’ demographic characteristics: age, sex, and comorbidities. Notably, comorbidities included a history of myocardial infarction (MI), congestive heart failure (CHF), peripheral vascular disease (PVD), acute cerebrovascular accidents or transient ischemic attacks (CVA), dementia (D), chronic obstructive pulmonary disease (COPD), connective tissue diseases, peptic ulcer disease (GI), liver disease (LD), diabetes mellitus (DM), hemiplegia, chronic kidney disease (CKD), solid tumors (ST), lymphoma, and leukemia (L). Comorbidities by the CCI and NEWS were considered as continuous variables and as categorical variables, respectively.

Length of stay (LoS) in ED, calculated as the difference from arrival time in the ED to the arrival time in the hospital ward, was considered as a continuous variable and as four time categories: ≤6 h, >6 to ≤12 h, >12 to ≤24 h, and >24 h. To account for differences in outcomes and care requirements among the elderly, age was considered as a continuous variable and as a categorical variable (≤85 vs. >85 years). Additional variables included ICD-9-CM diagnosis codes, categorized into traumatic and non-traumatic types.

In the logistic model, we also considered the COVID period (2020–2022) vs. non-COVID (2017–2019) as a variable to test for mortality due to the possible different case-mix characteristics of the two periods, the different years of presentation (from 2017 to 2022), months, and seasons (Table A3).

A generalized linear mixed regression model was developed, adjusting for factors such as age, sex, CCI ≥ 5, NEWS > 6, and trauma-related visits. In a second step, the model was expanded to include the ED length of stay (LoS) and time slots for ED visits (08:30 to 18:30 vs. 18:30 to 08:30). Selected variables were determined using stepwise regression and the recursive feature elimination technique. Results were presented as odds ratios (ORs) with two-sided 95% confidence intervals (95% CIs).

A mortality risk score was computed for each patient based on the coefficients derived from the logistic regression model, using variables entered through a stepwise procedure. The accuracy of this risk score was evaluated by the area under the receiver operating characteristic (ROC) curve. The optimal cutoff point (i.e., the value that maximizes both sensitivity and specificity) was determined using the Youden index [13].

To avoid ambiguity, no synthetic data were generated to handle missing data; thus, the analysis relied solely on complete cases. The statistical model was subsequently validated using event-number balancing techniques [14].

All analyses were performed using the Python programming language (version 3.10.12) in Jupyter Notebook. Key libraries included stats models (version 0.13.5), scipy (version 1.10.0), and scikit-learn (version 1.6.0).

## 3. Results

### 3.1. Participants

Our database included 20,009 patients aged ≥ 75 years that visited the ED and were admitted to hospital on the ordinary ward (88.5% of the initial cohort), divided into two groups: the EDOS group (3064 patients; 15.3%) and the Ward group (16,945 patients; 84.7%) (Figure 1).

### 3.2. Descriptive Data

The mean age of the study population was 85 years (median [IQR]: 80–89 years), with no significant differences between groups (Table 1). Males accounted for 45% of cases. The mean Charlson Comorbidity Index (CCI) was 5 (median [IQR]: 4–7), and 65.2% of cases had a CCI ≥ 5, with no differences between the two groups (Table 1). However, trauma-related visits were reported in 6219 cases (31.1%), occurring more frequently in the EDOS group. Additionally, the proportion of EDOS cases was higher during the COVID period (57.7%) than during the pre-COVID period (48.5%) (Table 1).

Most comorbidities were equally distributed in the two groups, except that CVA and solid tumor were more represented in the Ward group, while the history of hemiplegia was more frequent in the EDOS group (Table A1).

The mean NEWS score at arrival was 1 (SD 1.3), with fewer, but not significant, cases of NEWS > 6 recorded in the EDOS group (Table 1). The length of stay in the ED was longer in the EDOS group, particularly for cases in the 6 to 12 h (+2.4%), 12 to 24 h (+2.5%), and >24 h (+2.0%) stay categories (Table A2).

The most frequent ICD-9-CM main diagnosis codes were diseases of the circulatory system (390–459) (25.6% of cases), diseases of the respiratory system (460–519) (20.2%), trauma and poisoning (800–999) (13.5%), and diseases of the digestive system (520–579) (12.0%). Respiratory system, digestive system, genitourinary system, and infectious disease diagnoses were more prevalent in the EDOS group, while injuries, poisonings, and blood diseases were more common in the Ward group (Table 2).

The total number of cases was 3355 (16.8%) in 2017 and 3577 (17.9%) in 2022. However, a significant trend was observed in the EDOS group, with its proportion increasing from 13.8% in 2017 to 21.8% in 2022 (+36.9%; *p*-value for trend < 0.001, as shown in Table A3).

The analysis of case distribution across months and seasons of the year showed no significant differences, except for a reduction in the summer and an increase in winter in the proportion of subjects in the EDOS group. Additionally, a reversal of the ED/Ward group ratio during the COVID period compared with the pre-COVID period was observed, with a significant increase in the EDOS group during the COVID period (Table A3).

### 3.3. Outcome Data

Mortality was recorded in 3020 cases (15.1%). No differences were observed even after adjusting for available variables including sex, age, comorbidity index, severity indices at admission, and traumatic versus non-traumatic causes (for the original dataset, Chi-square statistic: 0.28, *p*-value: 0.59).

These results were consistent even after the application of case balancing techniques (Chi-square statistic: 0.3, *p*-value = 0.58) and further analyses testing for potential year, month, and seasonal effects as well as differences between the pre-COVID-19 and COVID-19 periods (Chi-square statistic: 4.17, *p*-value: 0.04; for the COVID-19 period, Chi-square statistic: 0.27, *p*-value: 0.6).

### 3.4. Main Results

**Figure 2:**
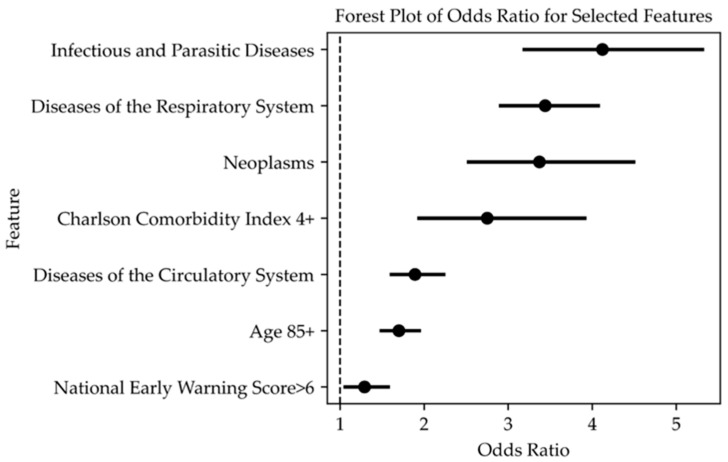
The forest plot of the selected variables entered into the logistic model for predicting mortality. Data are presented as odds ratios (OR) with 95% confidence intervals (95% CI); a *p*-value < 0.05 was considered significant. Variables are listed in order of importance from high to low.

Multivariable logistic regression was used as a predictive model with different sampling strategies (ranging from 0.1 to 1 in increments of 0.1) to evaluate the importance of features in predicting in-hospital mortality. A stepwise feature selection using a logistic model on randomly under-sampled data revealed that a set of variables entered the model for mortality (Table 3, Figure 2), except for the EDOS group, which was not included.

Variables not included in the logistic model were individual comorbidities, length of stay in the ED, overnight stay in the ED (EDOS), trauma-related visits, and factors such as different seasons, months, years, and the COVID period. Additionally, ICD-9-CM diagnosis codes related to neoplasms (140–239), endocrine, nutritional, metabolic diseases and immune system disorders (240–279), diseases of the blood and blood-forming organs (280–289), diseases of the nervous system and sense organs (320–389), mental and behavioral disorders (290–319), digestive system diseases (520–579), genitourinary system diseases (580–629), musculoskeletal system and connective tissue diseases (710–739), complications of pregnancy, childbirth, and puerperium (630–679), symptoms, signs, and laboratory findings (780–799), injury and poisoning, external causes (800–999), and external causes (E, V codes) were also excluded.

**Figure 3:**
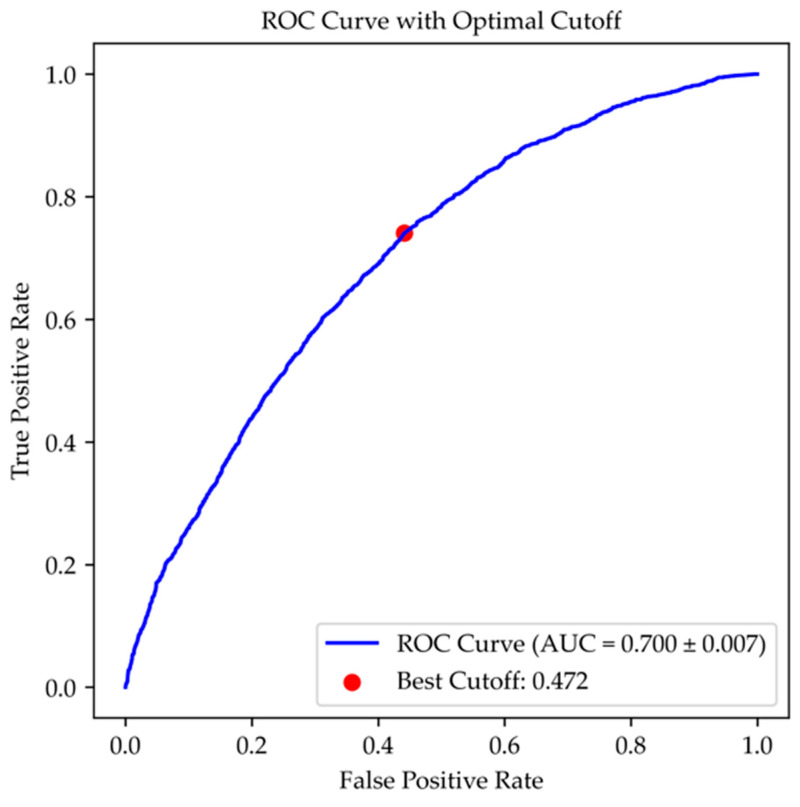
ROC plots of the risk score obtained from the logistic model, evaluating its ability to identify the increased mortality risk in patients admitted to conventional units.

The ROC curve for the mortality risk score, calculated based on the coefficients from the logistic regression, is shown in Figure 3. The accuracy in predicting the outcome was 0.700 ± 0.007, with a sensitivity of 74.1% and a specificity of 55.9% at the optimal cutoff point (score of 0.472), simultaneously maximizing both the sensitivity and specificity (Figure 3).

## 4. Discussion

### 4.1. Key Results

Our retrospective, monocentric study involves a review of the medical records of approximately 20,000 elderly patients (≥75 years) admitted to the hospital following evaluation in the ED. Mortality during hospitalization was associated with demographic and clinical variables but not with the time spent overnight in the ED waiting for a hospital bed.

Our findings are consistent with those of a similar Spanish multicenter study [8] that observed a trend—though not statistically significant—toward increased mortality (+12%) and prolonged hospital stays (+15–16%) among patients who remained in the ED overnight. The lack of statistical significance in that study may have been due to an insufficient sample size, as the minimum number of subjects needed to detect an effect could not be calculated due to the absence of prior studies.

Our results do not confirm those of a French study [6] that reported a significant increase in mortality (ranging from 39% to 50%) among patients staying overnight in the ED compared with those admitted during daytime hours, along with a non-significant increase in hospital length of stay (+5% to +20%).

The differences in results among these studies highlight the need to examine their methodological and contextual characteristics. The French study [6] analyzed a cohort of 1598 patients from 97 EDs over 3 days (December 2022) during a “triple pandemic” of respiratory viruses (influenza, respiratory syncytial virus, and COVID-19) and extreme overcrowding in healthcare facilities. In this context, 44% of patients spent the night in the ED, reflecting the strain placed on emergency systems during the global COVID-19 pandemic, particularly for vulnerable populations such as the elderly [15].

Conversely, the Spanish study [8] examined 3243 patients over a one-week period in April 2019, after the peak of respiratory infections and before the COVID-19 pandemic. While ED pressures remained high, they were less intense compared with those in the French study [6], with only 34% of patients (approximately 25% fewer) staying overnight in the ED.

While our study adhered to a similar methodology, it differed significantly in several aspects. First, it utilized retrospective data from a single center, with a much larger sample size (approximately 20,000 cases) and an extended observation period (6 years), compared with the Spanish (7 days) and French (3 days) studies. This long-term analysis allowed us to adjust the statistical model for the extraordinary overcrowding during the COVID-19 pandemic. Our findings conclude that, even after accounting for the potential effects of COVID-19, overnight ED stays were not, in themselves, associated with increased mortality among admitted patients.

Second, in our study, mortality was associated with several key variables including age over 85, a CCI ≥ 5, NEWS > 6 and specific diagnostic categories such as infectious diseases, respiratory diseases, and diseases of the circulatory system. Injury and poisoning as well as diseases of the blood and blood-forming organs were included in the logistic model but had negative coefficients.

In our model, overnight ED stay was not included as an independent variable. To ensure that the lack of effect was not due to the relatively low prevalence of overnight ED cases, we employed various sampling strategies (ranging from 0.1 to 1 in increments of 0.1) to balance the dataset and assess the impact of overnight ED stays on in-hospital mortality. Our results confirmed that overnight stays were not significantly associated with higher mortality, even when the frequency of ED overnight cases was comparable to that observed in the French study [6].

All three studies shared common methodological characteristics such as their retrospective design and a primary outcome of in-hospital mortality at 30 days. Each study adjusted the statistical model for demographic characteristics, vital signs at entry, comorbidities, ED waiting times, and ED length of stay. Notably, the Spanish study also included a dependency index (Barthel Index) [16], while the French study used the GIR score [17] to assess disability, but neither included diagnoses. In our study, to strengthen the hypothesis, we enhanced the logistic model with several key factors including the main diagnostic categories, the COVID-19 period, observation year, and seasonal periods.

It should be underlined that in a comparison between the main studies, the description of the characteristics of elderly patients who visited the ED usually included the number and type of comorbidities [18]. Among the features usually considered, cardiovascular diseases, diseases of the respiratory system, diabetes and digestive system diseases, with percentage differences in the different study contexts, were the most represented [6,8].

Furthermore, we calculated the accuracy of the statistical model, represented by the area under the curve (AUC), along with sensitivity and specificity data, which were not reported in the Spanish or French studies. Compared with the other studies, we provided additional information on the accuracy of our prognostic model, which demonstrated an AUC of 0.700 ± 0.007, with a sensitivity/specificity ratio favoring sensitivity at the optimal cutoff point (sensitivity 74.1%, specificity 55.9%).

The level of care provided during overnight ED stays may also influence the outcomes. While France and Spain share similar public healthcare systems, structural and technological differences as well as variations in inpatient care could have affected the results. In Italy and France, patients staying in EDs often receive only basic care on stretchers, without meals or specialized assistance. In contrast, Spanish authors [5] have reported that patients were provided with beds, meals, and beverages during their EDOS. Given these differences in the level of care, our findings suggest that the results are unlikely to be influenced by these factors.

It is important to underline that the French study has the merit of having further stimulated the discussion on the issue of long stays of elderly patients waiting for a bed in the ED [19]. Unfortunately, the difficulty of obtaining robust data to demonstrate this, due to the complexity of the phenomenon, may lead to the failure of several studies [20], despite professionals being sure that long stays in the ED waiting for a bed increases the risk of unfavorable events.

Our initial hypothesis was that the severe distress experienced by both patients and healthcare staff due to interruptions in care pathways (e.g., overnight ED stays) could adversely affect important outcomes such as mortality. Although it is clear that an overnight stay disrupts the clinical pathway of a patient requiring urgent hospitalization, our data do not support this hypothesis. Instead, the real question is not whether an overnight ED stay per se affects patient mortality, but rather what degree of severity influences the outcome.

The lack of significance in 30-day in-hospital mortality following an ED visit may be multifactorial. Contributing factors include heterogeneity among the studies themselves, along with various patient and system characteristics such as age, sex, comorbidities, triage severity score at arrival, type of disease, mode of arrival, daytime versus nighttime presentation, shift schedule, variation in hospital organization, adherence to clinical guidelines, the source of admission, and other relevant variables [21].

Nonetheless, given the results of our statistical model, it is reasonable to hypothesize that only in situations of severe ED overcrowding, where a high number of patients compromises the quality of care, may prognosis be influenced, leading to an increase in mortality.

### 4.2. Limitations

Several limitations of this study must be acknowledged. Firstly, although our study included a large number of cases over an extended period, the proportion of index cases—patients who experienced overnight stays in the EDOS—was lower than that reported in comparable studies. To address the potential impact of this discrepancy, we employed data balancing techniques to compare the percentage of EDOS cases with those from the French and Spanish studies [5,8]. However, these efforts did not yield significant findings, raising concerns about the reliability of simulated case scenarios based on data balancing techniques compared with real-world case studies. While random resampling was used, this approach could introduce uncertainty by excluding data potentially relevant to the outcomes [14].

Secondly, none of the studies incorporated a multidimensional geriatric assessment [22], which could be highly valuable for improving outcome prediction in the elderly population visiting the ED [23]. While the French and Spanish studies considered the GIR score and Barthel score [16,17], respectively, these tools only provided additional insights into the patients’ functionality.

Thirdly, our analysis did not include secondary outcomes such as the length of stay or complications during hospitalization. This omission may limit the ability to draw comparative insights between our results and those from other studies in the field.

Fourthly, in our study, we considered mortality at 30 days after ED visit as the main outcome. However, there is a possibility that some subjects who were discharged after a few days of hospitalization and unexpectedly died at home were not considered deceased, or even subjects who, due to complications during hospitalization, could have died after 30 days of hospitalization, the limit considered for the follow-up. However, we believe that the number of these cases is negligible and did not influence the results.

Fifthly, our database did not allow us to extract data or verify the impact of different quality of care provided at night compared with daytime stays. Only prospective studies could further investigate this aspect.

### 4.3. Interpretation

Our study did not find a relationship between mortality and ED overnight stays. This conclusion is based on an extensive 6-year dataset, though limited to a single-center study. The statistical model accounted for key prognostic factors including patient demographics, severity at presentation, case-mix variability across diagnoses, ED overcrowding levels as well as annual and seasonal trends.

Further investigation is needed to determine whether additional variables not included in the current models, such as multidimensional assessments [18], frailty index [24], and quality of care, could provide valuable insights into the prognostic outcomes of the aging patients.

### 4.4. Generalizability

In our study, the data were collected from a single-center, first-level ED within a local health agency, which may limit the generalizability of the findings. As a result, while the study spanned a six-year observation period, its conclusions may not be fully applicable to other facilities.

## Figures and Tables

**Table 1:** Baseline patient characteristics, including demographic variables, Charlson Comorbidity Index (CCI), National Early Warning Score (NEWS), trauma-related visits, and the COVID period, are reported as the number of cases (%) in order of importance of differences (means with 95% confidence intervals; 95% CI). A *p*-value < 0.05 was considered statistically significant.

	Total No.	EDOS Group No. (%)	Ward Group No. (%)	Difference (95% CI)	*p*-Value
**Patients**	20,009	3064 (15.3)	16,945 (84.7)	--	--
COVID period	9992 (49.9)	1768 (57.7)	8224 (48.5)	9.2 (7.3 to 11.0)	<0.001
Sex (males)	9044 (45.2)	1449 (47.3)	7595 (44.8)	2.5 (0.6 to 4.4)	0.011
Trauma-related	6219 (31.1)	1005 (32.8)	5214 (30.8)	2.0 (0.2 to 3.8)	0.025
CCI ≥ 5	13,046 (65.2)	2031 (66.3)	11,015 (65.0)	1.3 (−0.5 to 3.1)	0.171
Age > 85 years	9026 (45.1)	1398 (45.6)	7628 (45.0)	0.6 (−1.3 to 2.5)	0.532
NEWS > 6	2317 (11.6)	335 (10.9)	1982 (11.7)	−0.8 (−2.0 to 0.5)	0.224

**Table 2:** Patient characteristics concerning ICD-9-CM diagnosis codes, listed as the number of cases (%) in order of differences between the EDOS and Ward groups (means with 95% confidence intervals; 95% CI). A *p*-value < 0.05 was considered statistically significant.

Diagnosis (ICD-9-CM Code)	Total No.	EDOS Group No. (%)	Ward Group No. (%)	Difference (95% CI)	*p*-Value
Diseases of the Digestive System (520–579)	2403 (12.0)	458 (14.9)	1945 (11.5)	3.4 (2.1 to 4.8)	<0.001
Infectious and Parasitic Diseases (001–139)	1217 (6.1)	250 (8.2)	967 (5.7)	2.5 (1.4 to 3.5)	<0.001
Diseases of the Respiratory System 460–519)	4033 (20.2)	682 (22.3)	3351 (19.8)	2.5 (0.9 to 4.1)	0.002
Diseases of the Genitourinary System (580–629)	1467 (7.3)	281 (9.2)	1186 (7.0)	2.2 (1.1 to 3.3)	<0.001
Symptoms, Signs, and Laboratory Findings (780–799)	451 (2.3)	74 (2.4)	377 (2.2)	0.2 (−0.4 to 0.8)	0.514
Endocrine, Nutritional, and Metabolic (240–279)	439 (2.2)	74 (2.4)	365 (2.2)	0.2 (−0.3 to 0.9)	0.364
Congenital Malformations (740–759)	3 (0.01)	0	3 (0.1)	0 (−0.1 to 0.1)	0.461
Diseases of the Musculoskeletal System (710–739)	122 (0.6)	15 (0.5)	107 (0.6)	−0.1 (−0.4 to 0.2)	0.353
External Causes (E, V codes)	27 (0.1)	1 (0.03)	26 (0.2)	−0.17 (−0.2 to 0)	0.094
Mental Disorders (290–319)	220 (1.1)	29 (0.9)	191 (1.1)	−0.2 (−0.5 to 0.2)	0.377
Diseases of the Nervous System, Sense Organs (320–389)	263 (1.3)	35 (1.1)	228 (1.3)	−0.2 (−0.6 to 0.2)	0.363
Neoplasms (140–239)	972 (4.9)	134 (4.4)	838 (4.9)	−0.5 (−1.3 to 2.5)	0.175
Diseases of the Blood (280–289)	531 (2.7)	42 (1.4)	489 (2.9)	−1.5 (−2.0 to −1.0)	<0.001
Injury and Poisoning (800–999)	2697 (13.0)	306 (10)	2391 (14.1)	−4.1 (−5.3 to −2.9)	<0.001
Diseases of the Circulatory System (390–459)	5121 (25.6)	675 (22.0)	4446 (26.2)	−4.2 (−5.8 to −2.6)	<0.001

**Table 3:** The list of selected variables entered into the logistic model for mortality is reported in order of relevance. Data are presented as odds ratios (OR) with 95% confidence intervals (95% CI); a *p*-value < 0.05 was considered significant.

Independent Variables	OR (95% CI)	*p*-Value
Infectious and Parasitic Diseases (001–139)	3.51 (2.69 to 4.57)	<0.001
Diseases of the Respiratory System (460–519)	2.81 (2.35 to 3.36)	<0.001
Diagnosis Codes of Neoplasms (140–239)	2.66 (1.98 to 3.57)	<0.001
CCI ≥ 5	2.62 (1.84 to 3.71)	<0.001
Age > 85 years	1.72 (1.51 to 1.96)	<0.001
Diseases of the Circulatory System (390–459)	1.54 (1.29 to 1.84)	<0.001
NEWS > 6	1.27 (1.05 to 1.55)	<0.001

## Data Availability

The datasets generated during and/or analyzed during the current study are available from the corresponding author upon reasonable request.

## Abbreviations

The following abbreviations are used in this manuscript:

CCI	Charlson Comorbidity Index
CHF	Congestive heart failure
CKD	Hemiplegia, chronic kidney disease
COPD	Chronic obstructive pulmonary disease
CVA	Acute Cerebrovascular Accidents or Transient Ischemic Attacks
D	Dementia
DM	Diabetes mellitus
ED	Emergency department
EDOS	Emergency department overnight stay
GI	Connective tissue diseases, peptic ulcer disease
L	Lymphoma and leukemia
LD	Liver disease
MI	History of myocardial infarction
NEWS	National Early Warning Score
PVD	Peripheral vascular disease
ROC	Receiver operating characteristic
ST	Solid tumors

## Appendix A

**Table A1:** Baseline patient characteristics regarding the individual comorbidities at admission. Data are listed as the number of cases (%) in order of differences between the EDOS and Ward groups (means with 95% confidence intervals; 95% CI). A *p*-value < 0.05 was considered statistically significant.

Characteristic	Total No. (%)	EDOS Group No. (%)	Ward Group No. (%)	Difference (95% CI)	*p*-Value
**Patients**	20,009	3064 (15.3)	16,945 (84.7)	--	--
**History**					
CVA	3002 (15)	529 (17.3)	2473 (14.6)	2.7 (1.2 to 4.1)	<0.001
Solid Tumor	3583 (17.9)	605 (19.7)	2978 (17.6)	2.1 (0.7 to 3.7)	0.004
CKD	2452 (12.3)	408 (13.3)	2044 (12.1)	1.2 (0 to 2.6)	0.052
DM	3783 (18.9)	607 (19.8)	3176 (18.7)	1.1 (−0.4 to 2.6)	0.018
COPD	3293 (16.5)	523 (17.1)	2770 (16.3)	0.8 (−0.7 to 2.2)	0.321
CTD	1254 (6.3)	212 (6.9)	1042 (6.1)	0.8 (−0.2 to 1.8)	0.106
MI	1917 (9.6)	308 (10)	1609 (9.5)	0.5 (−0.6 to 1.7)	0.335
Leukemia	316 (1.6)	55 (1.8)	261 (1.5)	0.3 (−0.2 to 0.8)	0.298
Lymphoma	117 (0.6)	24 (0.8)	93 (0.5)	0.3 (−0.1 to 0.6)	0.117
PUD	543 (2.7)	80 (2.6)	463 (2.7)	−0.1 (−0.7 to 0.5)	0.703
LD	919 (4.6)	135 (4.4)	784 (4.6)	−0.2 (−1.0 to 0.6)	0.591
PVD	708 (3.5)	100 (3.3)	608 (3.6)	−0.3 (−1.0 to 0.4)	0.371
CHF	2056 (10.3)	307 (10)	1749 (10.3)	−0.3 (−1.4 to 0.9)	0.612
Dementia	4314 (21.6)	649 (21.2)	3665 (21.6)	−0.4 (−2.0 to 0.7)	0.580
Hemiplegia	1976 (9.9)	254 (8.3)	1722 (10.2)	−1.9 (−2.9 to −0.8)	0.165

**Table A2:** Number of subjects (%) of the EDOS group and Ward group regarding the different time categories. Data are reported as no. of cases (%) and the comparison between groups as the difference with 95% confidence intervals. A *p*-value < 0.05 was considered statistically significant.

Length of Stay	Total No. (%)	EDOS Group No. (%)	Ward Group No. (%)	Difference (95% CI)	*p*-Value
≤6 h	17,438 (87.2)	2491 (81.3)	14,947 (88.2)	−6.9 (−8.4 to −5.5)	<0.001
6 to ≤12 h	2316 (11.6)	417 (13.6)	1899 (11.2)	+2.4 (1.1 to 3.7)	<0.001
>12 to ≤24 h	194 (1.0)	95 (3.1)	99 (0.6)	+2.5 (1.9 to 3.2)	<0.001
>24 h	61 (0.3)	61 (2.0)	0	+2.0 (1.5 to 2.5)	<0.001

**Table A3:** Number of patients in the EDOS group and Ward group, categorized by year, month, and season during the study period. Data are reported as the number of cases (%), with differences expressed as the mean and 95% CI. Percent difference and *p*-values refer to a comparison between the EDOS group and the Ward Group.

	Total No. (%)	EDOS Group No. (%)	Ward Group No. (%)	Difference (95% CI)	*p*-Value
**Patients, No.**	20,009	3064 (15.3)	16,945 (84.7)	--	--
**Year**					
2017	3355 (16.8)	422 (13.8)	2933 (17.3)	−3.5 (−4.9 to −2.2)	<0.001
2018	3307 (16.5)	400 (13.1)	2907 (17.2)	−4.1 (−5.4 to −2.8)	<0.001
2019	3355 (16.8)	474 (15.5)	2881 (17.0)	−1.5 (−2.9 to −0.1)	0.037
2020	2728 (13.6)	461 (15.0)	2267 (13.4)	1.6 (0.3 to 3)	0.013
2021	3687 (18.4)	638 (20.8)	3049 (18.0)	2.8 (1.3 to 4.4)	<0.001
2022	3577 (17.9)	669 (21.8)	2908 (17.2)	4.6 (3.1 to 6.3)	<0.001
**Month**					
January	1848 (9.2)	298 (9.7)	1550 (9.1)	0.6 (−0.5 to 1.7)	0.309
February	1555 (7.8)	231 (7.5)	1324 (7.8)	−0.3 (−1.3 to 0.8)	0.602
March	1668 (8.3)	269 (8.8)	1399 (8.3)	0.5 (−0.5 to 1.6)	0.335
April	1679 (8.4)	290 (9.5)	1389 (8.2)	1.3 (0.2 to 2.4)	0.020
May	1720 (8.6)	263 (8.6)	1457 (8.6)	0 (−1 to 1.1)	0.978
June	1600 (8.0)	249 (8.1)	1457 (8.6)	−0.5 (−1.5 to 0.6)	0.773
July	1615 (8.1)	255 (8.3)	1360 (8.0)	0.3 (−0.7 to 1.4)	0.579
August	1741 (8.7)	230 (7.5)	1511 (8.9)	−1.4 (−2.4 to −0.3)	0.011
September	1673 (8.4)	233 (7.6)	1440 (8.5)	−0.9 (−1.9 to 0.2)	0.100
October	1794 (9)	277 (9.0)	1517 (9.0)	0 (−1 to 1.2)	0.875
November	1793 (9)	268 (8.7)	1525 (9.0)	−0.3 (−1.3 to 0.9)	0.652
December	1323 (6.6)	201 (6.6)	1122 (6.6)	0 (−1 to 0.9)	0.900
**Season**					
Summer	4956 (24.8)	734 (24.0)	4222 (24.9)	−0.9 (−2.6 to 0.7)	0.257
Fall	5260 (26.3)	778 (25.4)	4482 (26.5)	−1.1 (−2.7 to 0.6)	0.221
Spring	5067 (25.3)	822 (26.8)	4245 (25.1)	1.7 (0.1 to 3.5)	0.037
Winter	4726 (23.6)	730 (23.8)	3996 (23.6)	0.2 (−1.4 to 1.9)	0.771
